# New Possibilities of Substance Identification Based on THz Time Domain Spectroscopy Using a Cascade Mechanism of High Energy Level Excitation

**DOI:** 10.3390/s17122728

**Published:** 2017-11-25

**Authors:** Vyacheslav A. Trofimov, Svetlana A. Varentsova, Irina G. Zakharova, Dmitry Yu. Zagursky

**Affiliations:** 1 Faculty of Computational Mathematics and Cybernetics, Lomonosov Moscow State University, Leninskiye Gory, Moscow 119992, Russia; svarentsova@gmail.com (S.A.V.); zaharova@physics.msu.ru (I.G.Z.); zagurski@physics.msu.ru (D.Y.Z.); 2 Faculty of Physics, Lomonosov Moscow State University, Leninskiye Gory, Moscow 119992, Russia

**Keywords:** pulsed THz TDS, disordered structure, pulse with a few-cycles, computer simulation, spectral dynamics analysis method, integral correlation criteria, cascade mechanism, high energy level excitation, substance emission frequencies

## Abstract

Using an experiment with thin paper layers and computer simulation, we demonstrate the principal limitations of standard Time Domain Spectroscopy (TDS) based on using a broadband THz pulse for the detection and identification of a substance placed inside a disordered structure. We demonstrate the spectrum broadening of both transmitted and reflected pulses due to the cascade mechanism of the high energy level excitation considering, for example, a three-energy level medium. The pulse spectrum in the range of high frequencies remains undisturbed in the presence of a disordered structure. To avoid false absorption frequencies detection, we apply the spectral dynamics analysis method (SDA-method) together with certain integral correlation criteria (ICC).

## 1. Introduction

The detection and identification of hazardous chemical, biological and other substances by using remote sensing is one of the main current security problems. One of the ways to achieve this aim is by applying THz Time Domain Spectroscopy (TDS) because the THz radiation is non-ionizing and most common substances are transparent to it. Moreover, many hazardous substances have spectral fingerprints in the THz frequency range [1,2,3,4,5,6,7].

Substance identification is based, as a rule, on a comparison of the absorption frequencies of a substance under investigation with the corresponding frequencies from a database. Below we designate this identification method the standard THz TDS method. At present, this method is actively used not only for security applications, but also for the analysis and nondestructive inspection of a large number of materials [8,9], including multilayer composite materials [10,11,12,13,14,15], in food quality inspection [16,17,18,19], as well as for pharmaceutical and biomedical applications [20,21].

Despite the advantages of the THz radiation application, its application for substance detection and identification has some essential shortcomings [22]. Under real conditions, factors such as opaque packaging, inhomogeneity of the substance surface, atmospheric humidity and others affect significantly the THz spectroscopy results and distort the spectral features of the substance under investigation [23,24,25,26,27,28]. Thus, when observing a THz pulse reflected from a neutral material, we can detect false absorption frequencies, which are not inherent to this material but they may be wrongly detected as dangerous substances [29,30,31,32,33,34,35,36,37]. This serious disadvantage for the standard THz-TDS method and its physical mechanism understanding are challenging problems for the development of real detection systems.

That is why in the current paper, we study the influence of multi-layered covering on the spectral features of a substance as well as a way of avoiding the influence of a disordered structure on the pulse spectrum.

Below, using computer simulation we consider a substance layer placed between two coverings, which consist of linear layers with random dielectric permittivity. The incident THz Gaussian pulse with a few cycles falls on the substance, and then we analyze the spectral features of both the transmitted and reflected pulses. The first aim of this investigation is to demonstrate the possibility of the absorption (emission) frequency observation in both pulse spectra. Another aim consists in the finding of the emission frequencies corresponding to high energy level relaxation after their excitation due to the cascade mechanism. We show that these emission frequencies can be used for substance detection and identification. As a basis of our study, we use the Maxwell-Bloch equations [38,39,40].

In our opinion, the problem statement is a novel one and was not previously investigated. First, we provide an analysis of a few-cycle pulse interaction with a disordered structure. Note that in optics usually a laser pulse, containing many periods of electromagnetic field, falls on the photonic structure. In our case, the well-known transfer matrix method [41] for a description of the THz pulse interaction with the periodic structure is inapplicable, because we investigate short pulses with finite duration.

The second reason is the following: an active medium, which is placed inside the disordered structure, can be considered as an impurity of the periodical structure. In optics, similar impurities have sizes that are much less in comparison with sizes of the photonic crystal elements. In our case, the active medium length is comparable with or greater than the disordered structure length.

Third, we describe the characteristics of the active medium response to the THz pulse action in the framework of a matrix density formalism [42]. Thus, we take into account a time-dependent response of a medium. In optics, the temporal response (as well as resonant one) has not been analyzed yet.

In the current paper we show the spectral dynamics analysis method (SDA-method) efficiency and integral correlation criteria (ICC) ([29,30,31,33,34,35,36,37]) using for the detection and identification of a substance covered by such a disordered structure. This allows us to demonstrate a good chance to overcome the standard THz TDS method limitations. We discuss the spectral features of the signals transmitted through and reflected from the disordered layered structure by means of the SDA-method and with using specially developed ICC. In the present paper, we also deal with a multi-level energy medium, which is more relevant to real conditions and more complicated for substance detection.

It should be noted that the main feature of the integral correlation criteria consists in summing the correlation coefficients between spectral intensity evolution at a chosen frequency for the THz signal under investigation and the standard evolution from database. The summation is made during the time intervals of the THz signal analysis, which contain the main pulse or the first sub-pulse, or the remote part of this signal. This procedure is similar to the noise suppression in signal processing and allows us to decrease the influence of random fluctuation of correlation coefficient on a probability estimation of the standard substance spectral features presence in a THz signal under investigation.

Earlier the SDA-method was successfully applied for the identification of various neutral substances, explosives and drugs in the transmission and reflection mode [29,30,31]. In [33] the SDA method together with ICCs was applied for substance detection and identification using a high noise THz signal. In [34,35,36,37], the spectral properties of THz pulses measured at long distances of about 3.5 m were investigated by means of modified integral criteria. The essential limitations of the standard THz-TDS method were demonstrated in [30,31,34] as well. We note that the interaction of a THz pulse with a disordered layered structure was partly discussed in [34,37].

## 2. Physical Experiment with Thin Paper Layers

It should be noted that in [29] we investigated the spectral features of thin paper napkins in the THz frequency range. The sample consists of several layers of thin paper with total thickness of 5–7 mm and, obviously, it is an example of disordered layered structure in this frequency range. We call this signal Paper Layers for brevity. Below we briefly present the conditions of the physical experiment and show the appearance of the false absorption frequencies in the spectrum of the THz signal measured under real conditions.

**Figure 1:**
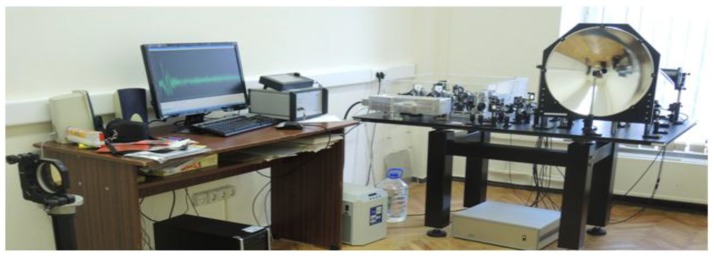
Experimental setup for THz signal measuring at long distance [29].

The measurements were carried out at a temperature of 18 °C and the relative humidity was about 50%. The distance between the parabolic mirror and the object was about 3.5 m. We exploited a THz spectrometer developed by Teravil Company (Vilnius, Lithuania). It uses a femtosecond fiber laser which generates the laser pulse with 1030 nm centre wavelength, 75 MHz repetition rate, with a pulse duration is about 80 fs. The spectral range of the spectrometer is 0.1–5.0 THz. SNR is better than 10^3^:1 (at 2 THz), 10^5^:1 (at 1 THz) and 10^6^:1 (at 0.4 THz). The spectral resolution is better than 10 GHz (fast scan), 2.5 GHz (combined mode of scan). The setup of the system can be found at http://www.ekspla.com. We used a parabolic mirror for focusing the THz beam on the object. Because the femtosecond fiber laser has average power of about 1 W and the laser beam splits many times, we use additional flat mirror behind the object. Therefore, our setup operates in reflection-transmission mode simultaneously. The experimental setup is shown in Figure 1.

**Figure 2:**
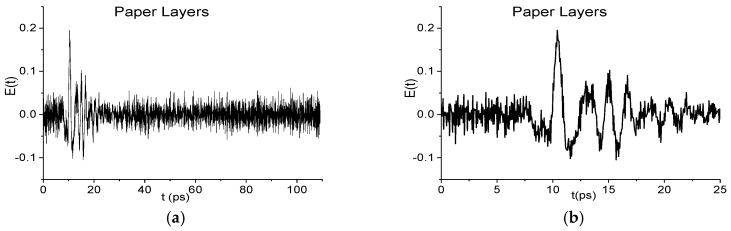
THz signal Paper Layers in the time interval *t* = [0, 110] ps. (**a**); [0, 25] ps. (**b**) [29].

**Figure 3:**
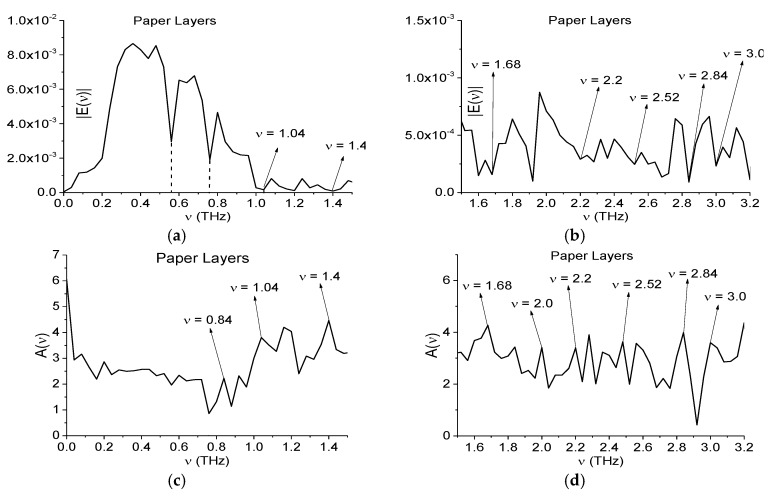
Fourier spectrum (**a**,**b**) and absorbance (**c**,**d**) of the main pulse for the signal Paper Layers in the frequency range *ν* = [0, 1.5] THz (**a**,**c**); [1.5, 3.2] THz (**b**,**d**) [29].

In Figure 2a the THz Paper Layers signal is presented in the time interval *t* = [0, 110] ps. The signal is very noisy and has a low SNR. In Figure 2b the main pulse of this signal is shown in the time interval *t* = [0, 25] ps. The Fourier spectrum of the Paper Layers main pulse is depicted in the frequency range *ν* = [0, 1.2] THz (Figure 3a), [1.1, 3.0] THz (Figure 3b). In Figure 3c,d the corresponding absorbance is depicted. Here, the absorbance *A*(ν) of a substance is defined as:

(1) $$A(\nu )=-\log_{10}(|E(\nu )|/|E_{REF}(\nu )|)$$

where |*E*(ν)|, |*E_REF_*(ν)| are the absolute values of spectral amplitudes of the measured and reference signals. The spectrum minima at the frequencies *ν* = 0.56, 0.76 THz in Figure 3a are caused by water vapor action [29].

It is obvious that the hazardous substances are absent in the paper napkins. However, the Fourier spectrum and absorbance demonstrate the spectral features of some of them. According to [2,3] the explosives RDX, HMX and PETN possess the following absorption frequencies: *ν* = 0.82, 1.05, 1.36, 1.54, 1.95, 2.19, 3.0 THz for RDX; *ν* = 1.78, 2.51, 2.82 THz for HMX; *ν* = 2.0, 2.16, 2.84 THz for PETN. One can see in Figure 3a,b the minima and in Figure 3c,d the maxima at frequencies *ν* = 0.84, 1.04, 2.0 THz, close to the absorption frequencies of RDX; the extremes at frequencies *ν* = 2.52, 2.84 THz, close to those of HMX (b), (d) and extremes at frequencies *ν* = 2.0, 2.84 THz, close to those of PETN (b), (d). The extremes *ν* = 1.4, 1.68 THz in Figure 3a–d are close to absorption frequencies of the illicit drug MDA [33].

Therefore, the standard THz-TDS method, based on the spectrum analysis only, is insufficient for the substance identification under real conditions because it detects the presence of many dangerous substances in a neutral sample. We stress that at the same time, the SDA-method together with ICC allows us to show the absence of dangerous substances in this sample and to confirm the presence of paper spectral features in it, see [29].

To explain the physical mechanism for the appearance of false absorption frequencies in the THz signal transmitted through or reflected from the multilayered sample in the papers [34,37] we performed a computer simulation using 1D Maxwell’s equations and matrix density formalism for a medium description. Below, we demonstrate the efficiency of the SDA-method for the detection and identification of a substance by using not only the absorption frequencies but also the emission frequencies. We consider a response of the substance covered by disordered structure.

## 3. The SDA Method Efficiency for the Detection of a Substance Covered by Disordered Structure

In this section, we focus our attention on the spectral intensity evolution in time at the chosen frequency of the signal transmitted through or reflected from the disordered layered structure.

The problem statement and the corresponding system of 1D Maxwell’s equations also are described in detail in [34,37]. All values (*t, ν, E(t), E(ν)*, etc.) used in the sections below, are dimensionless. Of course, we will discuss the efficiency of using the emission frequency [37] for the detection and identification of a substance. Applying the SDA-method and ICC we demonstrate the enhancement of information about the substance fingerprints. It is very important that the excitation of high energy levels due to the cascade mechanism occurs in the presence of a cover. Thus, the appearance of higher frequencies in the substance response spectrum is of great promising for the enhancement of THz –TDS efficiency.

### 3.1. SDA Method and Integral Correlation Criteria

In [29,30,31,33,34,35,36,37] we investigated the efficiency of using the integral correlation between the spectral line dynamics of the reflected or transmitted signal *E*(*t*) and the spectral line dynamics of the standard transmitted signal *e*(*t*) from database for the substance detection. For this purpose, we introduce the following notations. We denote the discrete set of spectral amplitude modulus for the standard transmitted signal *e*(*t*) at a chosen frequency *ν* as $e_{\nu }=\left\{\left|e_{\nu }\left(t_{m}\right)\right|\right\},\;m=1,\ldots ,M_{1}$. Note that the calculation of the spectral line (or spectral intensity) dynamics is described in a number of previous papers, for example, in [29]. The corresponding set of spectral amplitude modulus of the reflected (or transmitted) THz signal *E*(*t*) at the frequency *ν* is denoted as $E_{\nu }=\left\{\left|E_{\nu }\left(t_{m}\right)\right|\right\},\;m=1,\ldots ,M_{2}$, and its part with *M*_1_ components, which begins at the time moment *t_n_*, is denoted as $E_{\nu }{}^{\left(n\right)}=\left\{\left|E_{\nu }{}^{\left(n\right)}\left(t_{n+m}\right)\right|\right\}$. Here *M*_1_ and *M*_2_ are the numbers of time moments in the corresponding dynamics. These depend on the dynamics construction parameters—the window length *T* and its shift Δ along the signal. Accordingly to our previous investigations [29,30,31,33,34,35,36,37] we choose these parameters in the following way: the window length *T* = 2.8 ps., the window shift Δ = 0.2 ps.

Both sets $e_{\nu }=\left\{\left|e_{\nu }\left(t_{m}\right)\right|\right\}$ and $E_{\nu }{}^{\left(n\right)}=\left\{\left|E_{\nu }{}^{\left(n\right)}\left(t_{n+m}\right)\right|\right\}$ must be averaged at each step *t_n_* to avoid the influence of constant components of sets *e_ν_* and *E_ν_*^(*n*)^ on the correlation coefficient. Moving the set $e_{\nu_{1}}$ along the set $E_{\nu_{2}}$, we get at each time moment *t_n_* the correlation coefficient:

(2) $$c_{e,E}(t_{n})=\frac{\sum_{m=0}^{M_{1}-1}\left(|e_{\nu_{1}}(t_{m})-\bar{e_{\nu_{1}}}|)\cdot (|E_{\nu_{2}}(t_{m+n})-\bar{E_{\nu_{2}}}|\right)}{\left||e_{{}_{\nu_{1}}}-\bar{e_{\nu_{1}}}||\cdot ||E_{\nu_{2}}^{\left(n\right)}-\bar{E_{\nu_{2}}}|\right|},$$

where:

(3) $$\bar{e_{\nu_{1}}}=\sum_{m=0}^{M_{1}-1}\left|e_{\nu_{1}}(t_{m})\right|/M_{1},\;\bar{E_{\nu_{2}}}=\sum_{m=0}^{M_{1}-1}\left|E_{\nu_{2}}(t_{m+n})\right|/M_{1}.$$

Then, using this correlation coefficient *c_e,E_*(*t_n_*) we compute the following integral criteria for the substance detection and identification problem:

(4) $$C_{e,E}(t_{n})=\sum_{m=0}^{n}\left|c_{e,E}(t_{m})\right|,\;n=0,\ldots ,M_{2}-M_{1},$$

or:

(5) $$CW_{e,E}(t_{n})=\sum_{m=0}^{n}\left|c_{e,E}(t_{m})\right|w_{1}w_{2},\;n=0,\ldots ,M_{2}-M_{1},$$

where $w_{1}=w\left(\left|E\left(\nu_{1}\right)\right|\right)$, $w_{2}=w\left(\left|E\left(\nu_{2}\right)\right|\right)$ are the weight coefficients, which characterize the spectral brightness at each frequency *ν*_1_ and *ν*_2_ during the time interval of correlation computation. For example, they can be chosen as *w*_1_ = 1, *w*_2_ = 1 or $w_{1}=1/\left|E\left(\nu_{1}\right)\right|$, $w_{2}=1/\left|E\left(\nu_{2}\right)\right|$, or $w_{1}=1/({\left|E\left(\nu_{1}\right)\right|}^{2})$, $w_{2}=1/({\left|E\left(\nu_{2}\right)\right|}^{2})$. The last two pairs of weight coefficients take into account the substance absorbance at these frequencies and therefore, it is necessary to compute the spectrum brightness of the signal under analysis. Below we apply for identification a modification of the ICC (5), first introduced in [35]:

(6) $$CW1_{e,E}(t_{n})=\sum_{m=0}^{n}\left|c_{e,E}(t_{m})\right|w_{1},\;n=0,\ldots ,M_{2}-M_{1}.$$

This consists in using the weight coefficient $w_{1}=1/\left|e\left(\nu_{1}\right)\right|$. The advantage of this criterion is obvious. We use only the spectral brightness of the signal from database and therefore, both increase algorithm performance and decrease random fluctuation influence.

In certain cases, it is necessary to use one more criterion, which allows us to assess the similarity (or likeness) of two spectral line dynamics:

(7) $$L_{e,E}(t_{n})=\sum_{m=0}^{n}l_{e,E}^{}(t_{m}),\;n=0,\ldots ,M_{2}-M_{1},$$

where:

(8) $$l_{e,E}(t_{n})=1-\frac{\left||{\left(e_{\nu_{1}}-\bar{e_{\nu_{1}}}\right)}_{N}-{\left(E_{\nu_{2}}{}^{\left(n\right)}-\bar{E_{\nu_{2}}}\right)}_{N}|\right|}{\left||{\left(e_{\nu_{1}}-\bar{e_{\nu_{1}}}\right)}_{N}||+||{\left(E_{\nu_{2}}{}^{\left(n\right)}-\bar{E_{\nu_{2}}}\right)}_{N}|\right|},\;n=0,\ldots ,M_{2}-M_{1}.$$

The subscript *N* indicates that the corresponding variable in (8) is normalized, for example, in *L*_2_ norm.

In [29,30] we introduced the following definition for using the ICC: the frequency *ν* is detected in the signal under investigation, if the corresponding ICC calculated for the pair (*ν*, *ν*_1_) lies above all other ICC in the frequency detection range (FDR). Here the frequency *ν*_1_ belongs to a standard signal spectrum. As a rule, the boundaries of the FDR are extremes of the spectrum closest to the frequency under investigation. Vice versa, the frequency *ν* is not detected if there is at least one of other ICC that lies above the ICC corresponding to this pair in this frequency range.

### 3.2. Two Energy-Level Medium

In order to demonstrate the ability of effectively using the SDA-method together with ICC for detection and identification problems, in this section we briefly describe the computer simulation of a THz pulse interaction with a two-level medium, which possesses the single pronounced absorption frequency. Let us recollect that some substances (for example, RDX) possesses a very pronounced single absorption frequency [2,3]. Other frequencies do not cause a big dip in the spectrum of a THz pulse transmitted through the medium. Thus, there is a practical application of such investigation. The second remark is the following. This problem was partly discussed in [34], where we studied the interaction of a THz pulse with a two-energy level medium covered by disordered structure. However, it is necessary to provide its detailed analysis for understanding of the THz pulse interaction peculiarities with a multi-level medium. A further remark is made as follows. We have to repeat briefly a problem statement description to explain the results obtained.

**Figure 4:**
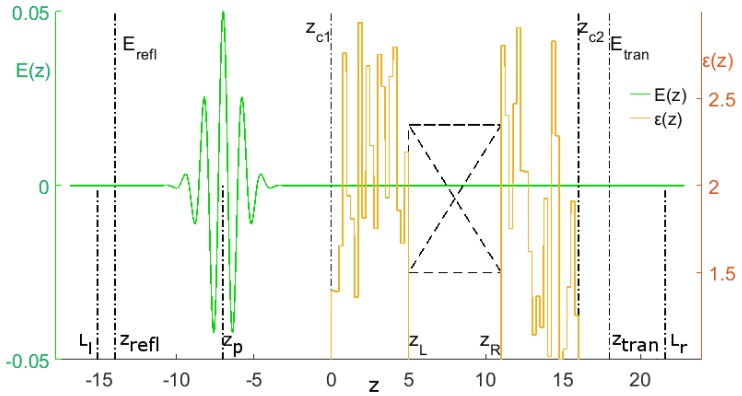
The scheme of the THz pulse interaction with covered substance. The left coordinate axis shows the electric field strength in dimensionless units. The dielectric permittivity of layers is shown on the right axis. The crossed rectangle depicts the active medium. Lines marked as *E_refl_* and *E_trans_* denote the coordinates at which the reflected and transmitted THz pulses are measured.

A scheme of the laser pulse propagation is shown in Figure 4. Several important coordinates are marked in the figure, namely the positions of the electric field detectors *E_refl_* and *E_trans_*, coordinates of the active substance faces *z_L_* and *z_R_*, and coordinates of the cover faces *z_c_*_1_ and *z_c_*_2_. The pulse propagates in vacuum, then transmits through both the left cover, medium, and finally, through the right cover. Then, the pulse exits into vacuum to the right. The pulse is partly reflected from various boundaries between layers and from the medium faces too. A certain part of the pulse energy is absorbed by the active medium. Generally speaking, a part of the absorbed energy can be emitted by the medium due to radiative transitions (emission) between excited energy levels. Reflected and transmitted pulses are detected near the left and right boundaries of the computational domain at the sections *E_refl_* and *E_trans_*. As a rule, these pulses may consist of a sequence of sub-pulses.

Electromagnetic field propagation is described by the 1D Maxwell’s Equations (7)–(14) in [37]. In [37], they were written in dimensionless variables. The dimensionless parameters of our computer simulation are defined in the following manner: the distance from the incident pulse centre to the left disordered structure is equal to 14 units; the length of the left and right disordered structures is equal to 5 units; the length of the resonant medium is equal to 5 units; the length of the right domain is equal to 3 units. Computation of the THz pulse interaction with a covered medium is provided during 100 dimensionless units. Pulse parameters are: the duration is 2 units, the pulse amplitude E_0_ is equal to unity; the current frequency of the wave packet ω_p_ is equal to 5. Two energy levels medium is described by the following parameters: the energy level transition frequency is equal to ω_12_ = 6. The dipole moment is d_12_ = 0.3, the transverse relaxation rate is γ_12_ = 0.05, and the longitudinal relaxation is W_mn_ = 0. Both the left and right disordered structures consist of the layers with random dielectric permittivity. The length of each of the structure layers is equal to 0.2 dimensionless units and their dielectric permittivities belong to the interval (1–1.7). It is randomly chosen with uniform distribution. Because below we analyze the resonant interaction of the THz pulse with a medium then the parameter χ (in Equation (13) [37],) is equal to zero (χ = 0).

**Figure 5:**
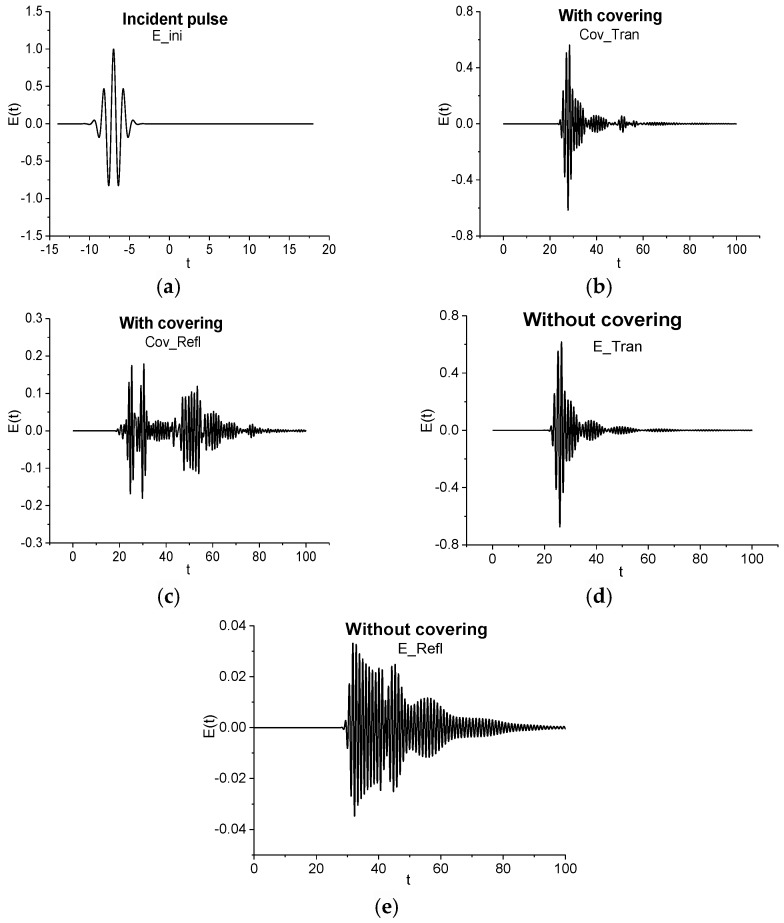
The incident pulse (E_ini) (**a**), pulses (Cov_Tran, E_Tran and Cov_Refl, E_Refl), correspondingly, transmitted through (**b**,**d**) and reflected from (**c**,**e**) the medium with (**b**,**c**) and without (**d**,**e**) covering.

Transmitted and reflected signals are averaged over 16, 32, 64 and 128 random realizations. The incident Gaussian pulse E_ini (a) (its shape is described by Equation (13), [37]) and one of the non-averaged realizations of the THz pulse after its interaction with the medium covered by disordered structure (or without covering) are shown in Figure 5a–c. We denote the transmitted signal as Cov_Tran (b) and the reflected signal as Cov_Refl (c). As one can see, the transmitted signal Cov_Tran (b) consists of the main pulse and several sub-pulses with smaller amplitudes, which follow the main pulse. The reflected signal Cov_Refl (c) contains more sub-pulses, which are caused by multiple reflections from boundaries of the disordered structure layers and the medium.

For comparison, the transmitted and reflected signals corresponding to the medium without covering are shown in Figure 5d,e. The notation E_Tran refers to the transmitted signal and, correspondingly, E_Refl—to the reflected signal. Comparing the signals E_Tran (d) and Cov_Tran (b), we see that the shape of the signal transmitted through the medium, covered by disordered structure, changes slightly. At the same time, the reflected signal Cov_Refl (c) has a more complex shape, containing several pronounced sub-pulses, in comparison with the signal E_Refl (e) reflected from a medium without covering. This feature of the reflected signal can be useful for the detection and identification of a substance under covering.

We stress once again that in our computer simulation, time *t* and frequency *ν* are dimensionless units, that is why in Section 3.2 and Section 3.3 we do not specify the time in picoseconds and frequency in terahertz.

#### 3.2.1. Transmitted Pulses

**Figure 6:**
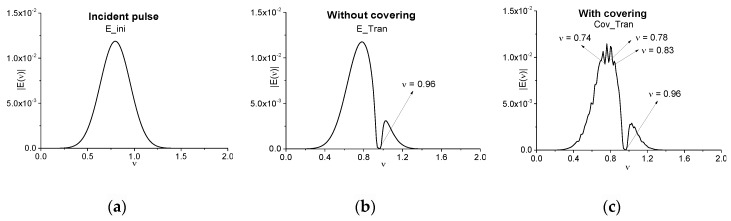
Fourier spectra of the incident pulse E_ini (**a**) and pulses E_Tran (**b**), Cov_Tran (**c**) transmitted through the medium without and with covering, correspondingly. The time interval of measurement is equal to *t* = [0, 100], the spectral resolution is equal to ∆ν = 0.01

In practice, it is very important to answer the following question: How does the thickness of a medium influence on the transmitted pulse spectrum? Let us remind ourselves that a pulse duration and the thickness of the medium in the case under consideration are of the same order. In Figure 6 the Fourier spectra of the incident pulse (a), of the pulse transmitted through the medium without covering (b), and with covering (c) are shown. In all cases (a)–(c) the spectral resolution is equal to ∆ν = 0.01. In both transmitted pulse spectra (b), (c) there is a minimum at the frequency *ν* = 0.96 = ω_12_/π, corresponding to the absorption frequency ω_12_ = 6 of the medium ([34]). However, if the medium is covered by a disordered layered structure, the transmitted signal spectrum (c) contains the additional minima at other frequencies *ν* = 0.74, 0.78, 0.83, which do not belong to the substance absorption frequencies. Therefore, they are false absorption frequencies.

**Figure 7:**
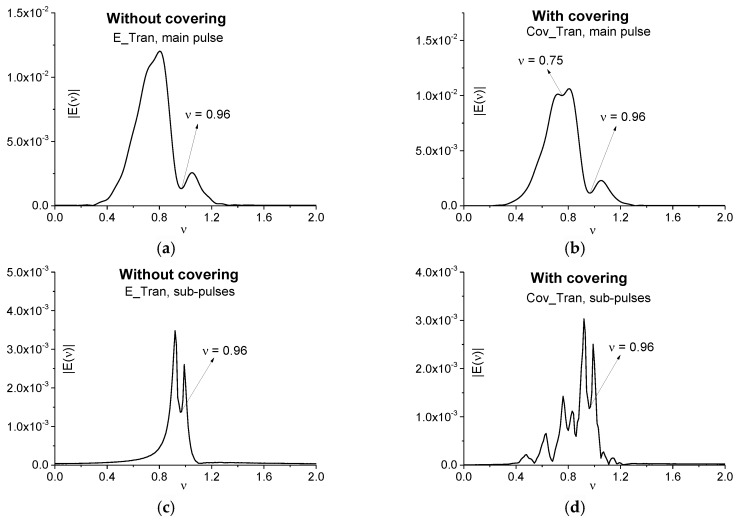
Fourier spectra of the main pulse (**a**), (**b**) and sub-pulse (**c**), (**d**) of the signal transmitted through the medium without (E_Tran) (**a**), (**c**) and with (Cov_Tran) covering (**b**), (**d**). The corresponding time intervals are equal to *t* = [0, 34] (**a**), [0, 36] (**b**), [34, 100] (**c**), [36, 100] (**d**). Spectral resolution is equal to Δν = 0.01.

In many cases for substance detection, it is necessary to provide the spectrum analysis for the main pulse and sub-pulses separately. Therefore, Figure 7a,b show the Fourier spectra of the main pulses transmitted through the medium without covering (E_Tran) (a) and with it (Cov_Tran) (b) in the time intervals *t* = [0, 34] (a), *t* = [0, 36] (b), correspondingly. In the spectrum (a) there is a single minimum at the absorption frequency *ν* = 0.96. One can also observe the inflection point at the frequency *ν* = 0.75. Its appearance is due to the medium finite thickness influence. In (b) (the medium with covering) the spectral intensity minimum at the frequency *ν* = 0.96 preserves, but we can also see the single false absorption frequency *ν* = 0.75.

In the sub-pulse spectrum corresponding to the pulse propagation through the medium without covering (c) one can observe the single minima at the absorption frequency *ν* = 0.96. However, several false absorption frequencies together with the substance absorption frequency *ν* = 0.96 appear for the pulse transmitted through the covered medium (d). Comparing the spectra (c) and (d), one can conclude that the false absorption frequencies appearance in the spectrum (d) is caused by the influence of covering mainly. Thus, the absorption frequency is seen more clearly in the spectrum of the first sub-pulse.

**Figure 8:**
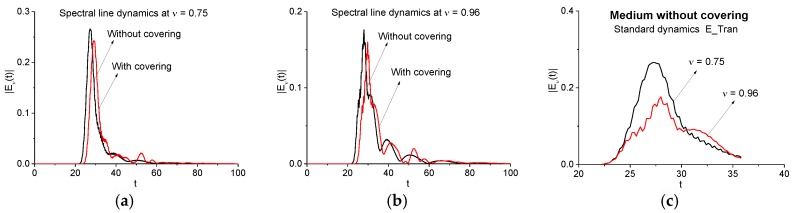
Spectral line dynamics at the frequencies *ν* = 0.75 (**a**), 0.96 (**b**) for the pulses transmitted through the medium without covering (E_Tran) and with it (Cov_Tran) in the full time interval *t* = [0, 100]. Spectral line dynamics of the standard pulse E_Tran calculated at the frequencies *ν* = 0.75, 0.96 using the time interval *t* = [20, 35] (**c**).

To detect and identify the substance, we apply the SDA-method and, therefore, we need to use the spectral line dynamics at chosen frequencies. To underline an influence of covering on time-dependent spectral intensity at the substance absorption frequency, we show in Figure 8 the spectral intensity evolution in time for the signals transmitted through the medium without covering (E_Tran) and with covering (Cov_Tran). They are calculated for the frequencies *ν* = 0.75 (a), 0.96 (b) in the full time interval *t* = [0, 100]. One can see the time delay between the spectral amplitudes (a), (b). Obviously, it is caused by the optical density of covering that slows down the pulse velocity. Another influence of covering becomes apparent in the maximal spectral intensity decreasing for the Cov_Tran pulse in (a), (b). It means that the most part of the spectral energy at these frequencies will be concentrated in the reflected signal and, therefore, a detection of this substance frequency will be more effective.

Below we will use the spectral line dynamics of the signal E_Tran calculated at the frequencies *ν* = 0.75, 0.96 in the time interval *t* = [20, 35] (c) containing the main pulse as the standard spectral dynamics. The dynamics at the frequency *ν* = 0.75 will be used in order to show that this frequency is a false one in the Cov_Tran main pulse spectrum (see Figure 7b) by means of ICC.

Below we use several ICC for the detection and identification of substances. The criterion *CW_e,E_* (5) requires spectral intensities at the chosen frequency for a signal under investigation and a standard signal from database during the time intervals under analysis. They are used as so called “weight coefficients”. In contrast to the criterion *CW_e,E_*, the ICC *CW1_e,E_* (6) uses only the spectral intensity of the standard signal from database. This allows a reduction of the signal random fluctuation influence on detection probability. The other ICC *C_e,E_* (4) and *L_e,E_* (7) allow us to assess the integral correlation and “likeness” of two spectral line dynamics. It is important that the ICC *C_e,E_* is calculated without using the weight coefficients, so the contrast of detection when used is usually less than when ICC *CW_e,E_* is used.

**Figure 9:**
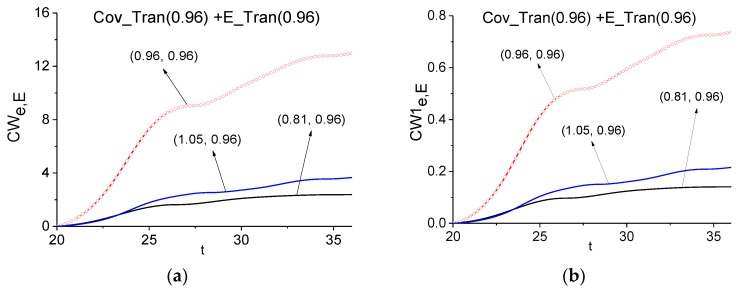
Time-dependent ICC *CW_e,E_* (**a**), *CW1_e,E_* (**b**) calculated at the frequency *ν* = 0.96 in the time interval *t* = [20, 36] for the pair of pulses transmitted through the medium with covering (Cov_Tran—a signal under investigation) and without covering (E_Tran—a standard signal).

First, we detect the frequency *ν* = 0.96 in the pulse transmitted through the medium with covering (Cov_Tran) by using the ICC *CW_e,E_* and *CW1_e,E_*. In Figure 9 these ICC are calculated in the time interval *t* = [20, 36] containing the main pulse. The corresponding FDR is equal to *ν* = [0.81, 1.05]. We see that the frequency *ν* = 0.96 is detected as the absorption frequency of the medium at using both criteria. The detection contrast slightly increases when using the ICC *CW1_e,E_*.

As we see in Figure 6c and Figure 7b, there is an absorption frequency *ν* = 0.75 in the spectrum of the pulse transmitted through the medium covered by disordered structure. We show that this frequency is a false absorption frequency. With this aim, we use the E_Tran spectral line dynamics at this frequency during the main pulse as a standard one.

**Figure 10:**
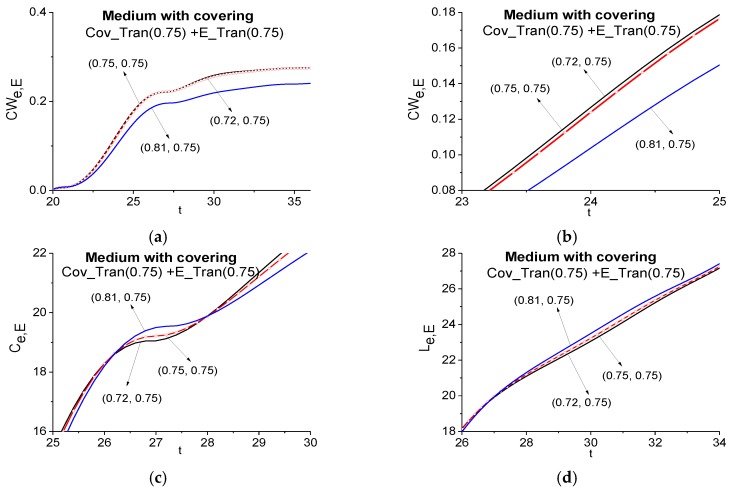
Time-dependent ICC *CW_e,E_* (**a**), (**b**), *C_e,E_* (**c**), *L_e,E_* (**d**) calculated at the frequency *ν* = 0.75 in the time intervals *t* = [20, 36] (**a**), [23, 25] (**b**), [25, 30] (**c**), [26, 34] (**d**) for the pair of pulses transmitted through the medium with covering (Cov_Tran—a signal under analysis) and without covering (E_Tran—a standard signal).

In Figure 10 the ICC *CW_e,E_* evolution is shown at the frequency *ν* = 0.75 for the Cov_Tran main pulse during the time interval *t* = [20, 36] (a). The lines corresponding to the frequency pairs *ν* = (0.75, 0.75) and *ν* = (0.72, 0.75) almost coincide. Therefore, in (b) we present the same criterion evolution in the enlarged scale for the time interval 23 < *t* < 25. Here one can clearly see that the frequency *ν* = 0.75 is not detected as the absorption frequency of the medium in the signal Cov_Tran transmitted through the medium with covering because the corresponding ICC is not the top-most in the FDR.

Moreover, in Figure 10c,d the integral criteria *C_e,E_* (c) and *L_e,E_* (d) also do not detect this frequency *ν* = 0.75. Thus, we have shown that the frequency *ν* = 0.75 is a false absorption frequency, which was induced by the covering of the substance in the Cov_Tran signal.

In the same way, it is possible to show the absence of spectral features of dangerous substances, for example, of explosives, in the signal transmitted through the covered medium (Cov_Tran). For this purpose, we use the THz signal transmitted through the tablet containing 10% RDX and 90% PE in ambient air (we denote it as RDX_Air signal) as a standard one. The measurement was carried out during the time interval 0 < *t* < 10 ps. at the room temperature 22 °C, and relative humidity of about 50% at the Center for Terahertz Research, Rensselaer Polytechnic Institute, Troy, NY, USA. The well-known absorption frequency of RDX is *ν* = 0.82 THz [2,3] and it is the closest to the minimum of Cov_Tran spectrum (Figure 7b) at the frequency *ν* = 0.75. The RDX_Air spectral line dynamics at *ν* = 0.82 THz is presented in [30], therefore, we use it as a standard one for detecting the absorption frequency of RDX.

**Figure 11:**
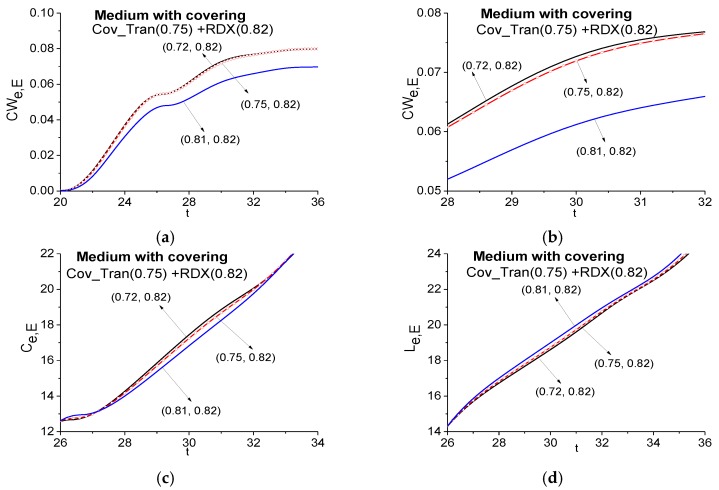
Time-dependent ICC *CW_e,E_* (**a**), (**b**), *C_e,E_* (**c**), *L_e,E_* (**d**) calculated at the frequency *ν* = 0.75 in the time intervals *t* = [20, 36] (**a**), [28, 32] (**b**), [26, 36] (**c**), (**d**) for the signal transmitted through the medium with covering (Cov_Tran—a signal under analysis) and the RDX_Air signal as a standard one.

As an example, in Figure 11 the ICC *CW_e,E_* evolution is shown in the time interval *t* < 36. It does not detect the frequency *ν* = 0.75 as an absorption frequency of the explosive RDX in the Cov_Tran spectrum. The lines corresponding to the frequency pairs *ν* = (0.75, 0.82) and *ν* = (0.72, 0.82) almost coincide in (a). Therefore, in (b) the same criterion evolution is presented in the enlarged scale in the time interval 28 < *t* < 32, and we see that the frequency *ν* = 0.75 is not detected as RDX absorption frequency. Additionally, in (c), (d) the ICC *C_e,E_* and *L_e,E_* also do not show a presence of the RDX spectral feature at the frequency *ν* = 0.75 in the signal Cov_Tran.

**Figure 12:**
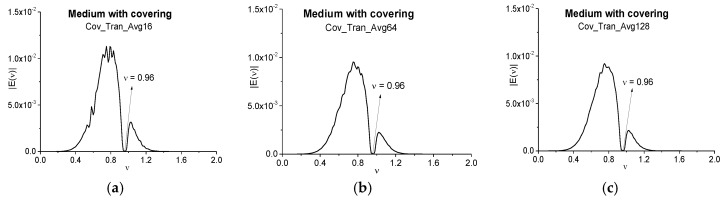
Fourier spectra of the averaged signals Cov_Tran_Avg16 (**a**), Cov_Tran_Avg64 (**b**), Cov_Tran_Avg128 (**c**) transmitted through the medium with covering and calculated in the time interval *t* = [0, 100].

As mentioned above, the signals transmitted through the disordered layered structure are averaged over a number of random realizations. This operation is usually used in experiments in order to decrease a noise influence. Therefore, it is of interest in practice to investigate an influence of random realizations number on the false absorption frequency appearance. With this aim in Figure 12 the signal Fourier spectra averaged over 16 (a), 64 (b), and 128 (c) realizations are depicted. They are calculated in the time interval *t* = [0, 100] with the spectral resolution ∆ν = 0.01. The spectrum minimum at the medium absorption frequency *ν* = 0.96 is present in all spectra (a)–(c), but other spectrum minima in the frequency interval *ν* = [0.4, 0.9] change their positions in dependence of the average number. Moreover, the number of spectra minima decreases with increasing the average number. These two features are an additional argument for elimination of these frequencies from consideration at analysis of the substance spectral properties, and their appearance in the spectra is due to the disordered structure influence. The same situation is observed in the main pulse spectra of the averaged signal.

Thus, covering results in the false absorption frequencies appearing in the spectrum of the pulse transmitted through a covered medium at a resonant interaction of a THz pulse with this medium. Nevertheless, using the ICC’s *CW_e,E_*, *C_e,E_* and *L_e,E_* allows us to eliminate from consideration the false absorption frequency *ν* = 0.75, which appears in the main pulse spectrum of the signal Cov_Tran transmitted through the medium with covering. As well, the ICC’s *CW_e,E_*, *C_e,E_* and *L_e,E_* show the absence of RDX absorption frequencies in the Cov_Tran main pulse. When averaging the signal Cov_Tran, the number of false absorption frequencies decreases with increasing the average number and they do not preserve their positions.

#### 3.2.2. Reflected Signals

**Figure 13:**
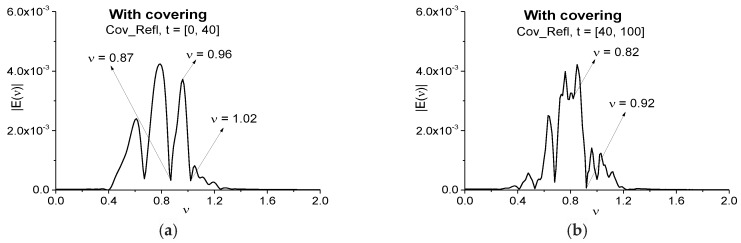
Fourier spectra of the signal reflected from the covered medium (Cov_Refl) and calculated in the time intervals *t* = [0, 40] (**a**) and *t* = [40, 100] (**b**).

Below we investigate the spectral features of the pulse reflected from the resonant two energy-level medium with and without covering. In the analysis of the pulse reflected from the substance, it is useful to consider the main pulse and sub-pulses spectra separately for the substance detection and its identification. In most cases, the main pulse spectrum does not contain useful information about the substance absorption frequencies. Therefore, Figure 13 shows the Fourier spectra of the signal reflected from the covered medium (Cov_Refl) in the time intervals *t* = [0, 40] (a), [40, 100] (b). They correspond to the main reflected pulse (a) and the sub-pulses (b). The spectra are calculated with the spectral resolution Δν = 0.01. It should be noted that the minimum close or equal to the medium absorption frequency *ν* = 0.96 is absent in the main reflected pulse spectrum (a), and at this frequency we observe maximum in (a). Thus, in the time interval *t* = [0, 40] we cannot identify a medium by using the standard method of the detection and we do not apply the SDA method because of this absorption frequency absence.

At the same time, in the time interval [40, 100] (b), one can see the spectrum minimum at the frequency *ν* = 0.92. In our opinion, we observe in (b) the absorption frequency shifting from the frequency *ν* = 0.96 to the frequency *ν* = 0.92 due to the influence of covering. Therefore, we will use this frequency for the detection of the medium covered by disordered structure at using the spectral line dynamics of the signal E_Tran, calculated at the frequency *ν* = 0.96 as a standard one.

**Figure 14:**
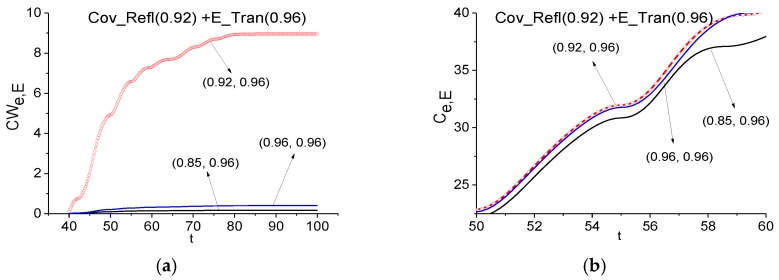
Evolution of the ICC *CW_e,E_* (**a**), *C_e,E_* (**b**) calculated for the frequency *ν* = 0.92 using the Cov_Refl signal and the E_Tran signal as a standard one.

The corresponding evolution of the ICC’s *CW_e,E_* (a) and *C_e,E_* (b) are shown in Figure 14. The ICC *C_e,E_* is shown in the enlarged scale in order to demonstrate the difference between the lines for the frequency pairs *ν* = (0.92, 0.96) and *ν* = (0.96, 0.96), which are close to each other. One can see that the frequency *ν* = 0.92 is detected for both criteria in the FDR *ν* = [0.85, 0.96]. Therefore, the absorption frequency of the medium is found in the travelling part (*t* > 40) of the signal Cov_Refl reflected from the covered medium, which does not contain the main pulse.

**Figure 15:**
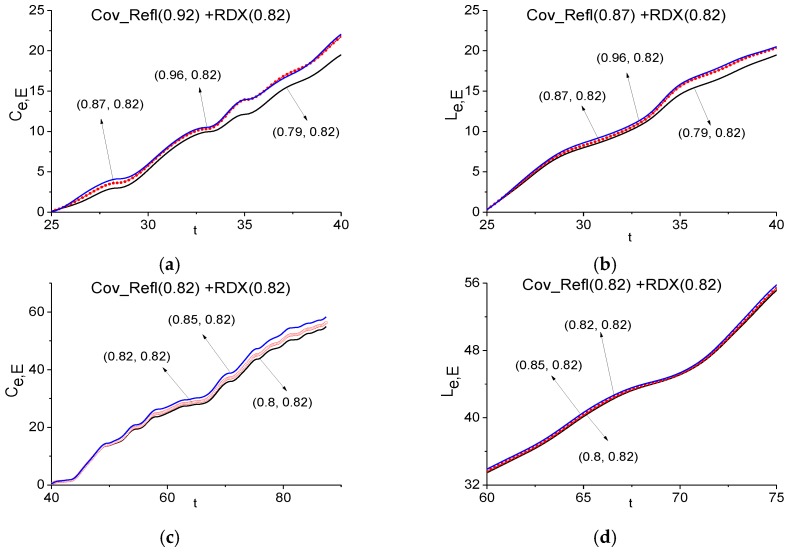
Time-dependent ICC *C_e,E_* (**a**), (**c**) and *L_e,E_* (**b**), (**d**) calculated for the frequencies *ν* = 0.87 (**a**), (**b**) and 0.82 (**c**), (**d**) during the time intervals *t* = [25, 40] (**a**), (**b**), [40, 90] (**c**), (**d**) for the signal reflected from the medium with covering (Cov_Refl) and the RDX_Air signal as a standard one.

In Section 3.2.1, we showed the absence of the RDX spectral features in the signal transmitted through such a medium covered by a disordered structure. In practice, it is very important to provide a similar investigation using the reflected signal. Moreover, in the spectra of the main pulse and the travelling part of the signal Cov_Refl (see Figure 13) one can observe the minima at the frequencies *ν* = 0.87 (a) and 0.82 (b), which are close or equal to the RDX absorption frequency *ν* = 0.82. Therefore, we can believe that RDX is present in the sample under investigation. In Figure 15 we show the ICC *C_e,E_* and *L_e,E_* evolution and see that this frequency cannot be detected as the RDX absorption frequency in the signal Cov_Refl, so the ICC is calculated in the time interval *t* = [25, 40] (a), (b), which contains the non-zero part of the Cov_Refl main pulse and in the time interval *t* = [40, 90] (c), (d), which does not contain the main pulse.

The likeness criterion *L_e,E_* is shown in (d) at the enlarged scale *t* = [60, 75] in order to demonstrate the difference between the lines, which also does not show the RDX absorption frequency presence in the Cov_Refl signal.

**Figure 16:**
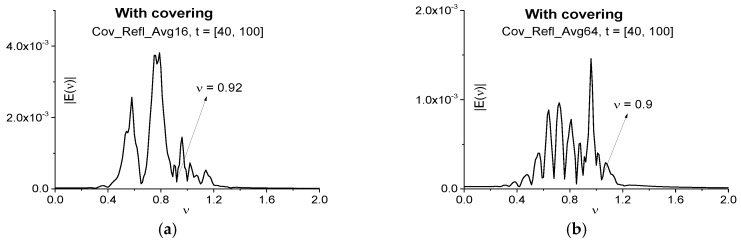
Fourier spectra of the signals Cov_Refl_Avg16 (**a**) and Cov_Refl_Avg64 (**b**) reflected from the covered medium in the time interval *t* = [40, 100] not containing the main pulse.

To illustrate the influence of a number of random realizations on the averaged pulse spectrum, we show in Figure 16 the Fourier spectra of signals Cov_Refl_Avg16 (a) and Cov_Refl_Avg64 (b) reflected from the medium with covering and calculated in the time interval *t* = [40, 100], not containing the main pulse. We consider this time interval as the medium absorption frequency *ν* = 0.96 is absent in the spectrum of the main reflected pulse (*t* = [0, 40]). The signals are averaged over 16 (a) and 64 (b) random realizations, correspondingly. As above, the spectral resolution is ∆ν = 0.01. Essentially that the spectral minimum at the frequency *ν* = 0.92 takes place in (a). However, in (b) the similar minimum occurs at the close frequency *ν* = 0.9, and moreover, the number of minima increases in (b). It is important that in both cases (a) and (b) the ICC *CW_e,E_*, *C_e,E_* and *L_e,E_* also detect the frequencies *ν* = 0.92 (a) and *ν* = 0.9 (b) in the signals Cov_Refl_Avg16 and Cov_Refl_Avg64 reflected from the medium with covering as the absorption frequencies of the uncovered medium (not shown). Minima shifting is caused by the influence of covering.

Therefore, we demonstrate the false frequencies appearing in the spectrum of the reflected pulse at a resonant interaction of a THz pulse with the covered medium. The ICC’s *C_e,E_* and *L_e,E_* show the absence of RDX absorption frequencies in different time intervals, which contain the non-zero part of the reflected signal Cov_Refl main pulse and do not contain it. Signal averaging over certain random realizations of layered structure dielectric permittivities cannot remove all false absorption frequencies, but their spectral position changes for different number of random realizations. On the other hand, the ICC’s *CW_e,E_* and *C_e,E_* detect the true absorption frequency of a medium in the travelling part of the reflected signal Cov_Refl, which does not contain the main pulse. It is should be noted that the covering influence is also manifested in the frequency shift from the frequency *ν* = 0.96 to the frequency *ν* = 0.92 in the time interval, which does not contain the Cov_Refl main pulse.

### 3.3. Three Energy-Level Medium

The main aim of this section consists in demonstration of emission at the high frequency, which appears due to the cascade mechanism of high energy level excitation, as well for emission frequency using for the substance detection and identification. It should be stressed that in this case the parameter χ ≠ 0 (namely, χ = 3, see Equation (11) [37]), the medium exhibits both resonant and non-resonant responses to the THz pulse interaction with medium. The physical and simulation parameters for this interaction are given in the Tables 1 and 2 [37]. The signal notations are the same as in the Section 3.2. First, we consider the signal transmitted through a medium.

#### 3.3.1. Signals Transmitted through the Medium

In this sub-section, we briefly repeat a part of the spectra analysis for the signals transmitted through the medium without and with covering, which was done in [37]. It is necessary for substance detection and identification by means of ICC in the most effective way at using the medium emission frequency in the different time intervals.

**Figure 17:**
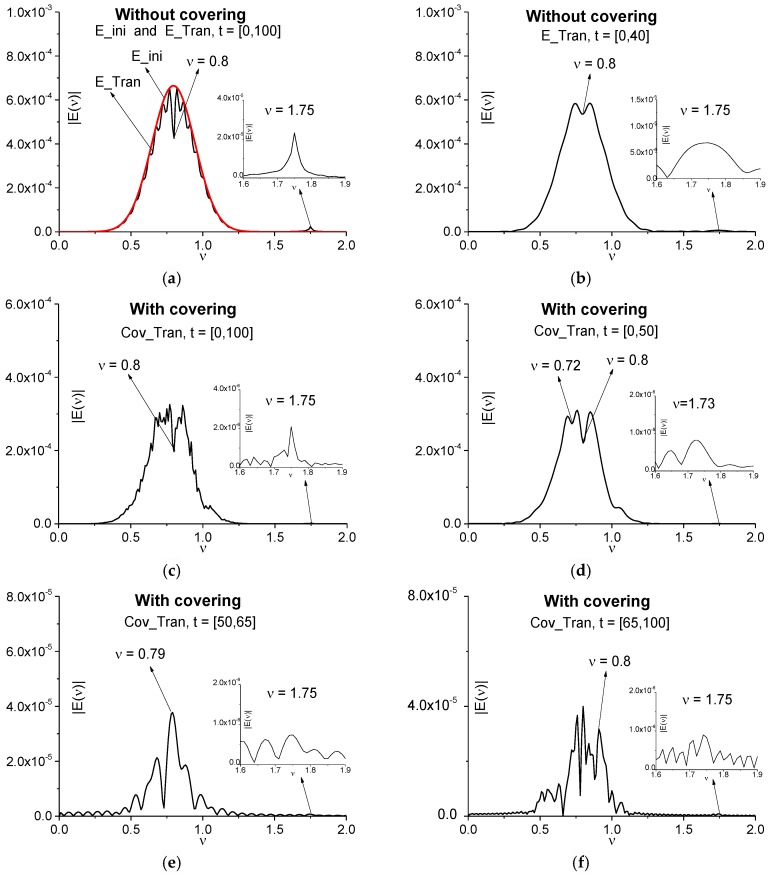
Fourier spectra of the signals transmitted through the uncovered medium (E_Tran) (**a**), (**b**) and medium with covering (Cov_Tran) (**c**)–(**f**) in the time intervals *t* = [0, 100] (**a**), (**c**), [0, 40] (**b**), [0, 50] (**d**), [50, 65] (**e**), [65, 100] (**f**).

The Fourier spectra of the signal E_Tran (a medium without covering) and the signal Cov_Tran (a medium with covering) are depicted in Figure 17. The pulse spectra are calculated in the time interval *t* = [0, 100], containing the full signals (a), (c); in the time intervals *t* = [0, 40] (b), [0, 50] (d), containing the main pulses only, and in the time intervals *t* = [50, 65] (e), [65, 100] (d), containing the subsequent sub-pulses. The computation is made with the spectral resolution ∆ν = 0.01. For comparison, in (a) the Fourier spectrum of the incident pulse E_ini is depicted. We see that all spectra (a)–(d) possess the pronounced spectral minimum at the frequency *ν* = 0.8 corresponding to the medium absorption at the circular frequency ω = 5.0 (see Table 2 [37]), where ω = 2πν. However, in the spectra of the transmitted signal E_Tran (a) as well as for the signal Cov_Tran (c), calculated in the full time interval *t* = [0, 100], there are additional minima. As to the main pulses spectra (c), (d), the additional minima are absent in (b) (a medium without covering), but we do see the additional minimum appearance in (d) at the frequency *ν* = 0.72 (a medium with covering).

Let us note that the additional minima in the spectrum for the signal transmitted through the non-covered medium (E_Tran) (a) are caused by the interference of the subsequent sub-pulses formed due to multi-reflection from the medium faces. Covering of the medium (Cov_Tran) (c)–(f) leads to additional modulation of the spectrum because of THz radiation reflection from the layers of a disordered structure.

The essential feature of the Figure 17 is the emission frequency presence in all spectra. We see the spectrum maxima at the frequencies *ν* = 1.75 (a), (b), (c), (e), (f) and 1.73 (d) corresponding to the medium emission at the circular frequency ω = 11.0. This frequency appears due to two stages of the THz pulse interaction with a medium. The first stage is the third energy level excitation of a medium due to cascade mechanism. The second one is a relaxation of molecules from this excited energy level to the ground energy level, which leads to emission at this frequency. It can be used for substance detection and identification.

To compare the main pulse analysis efficiency with the sub-pulse analysis efficiency for THz system operating in transmission mode, in (e), (f) the Cov_Tran signal spectra are shown for the time intervals *t* = [50, 65] (e) and *t* = [65, 100] (f), which contain only the additional sub-pulses of the transmitted signal. Comparing these figures, we can conclude that at the transmitted signal analysis, the main pulse consideration is more preferable.

Nevertheless, we see that the spectrum used only for the detection and identification of a substance under a covering is insufficient because of many false absorption frequencies present. Therefore, we need to apply the ICC for this aim and analyze the medium emission at the frequency *ν* = 1.75. In this connection, we show a role of the main pulse for the identification problem in Figure 17. Note that in (b) and (d) a pronounced spectral maxima at the frequency *ν* = 1.75 (b) and *ν* = 1.73 (d) are seen.

**Figure 18:**
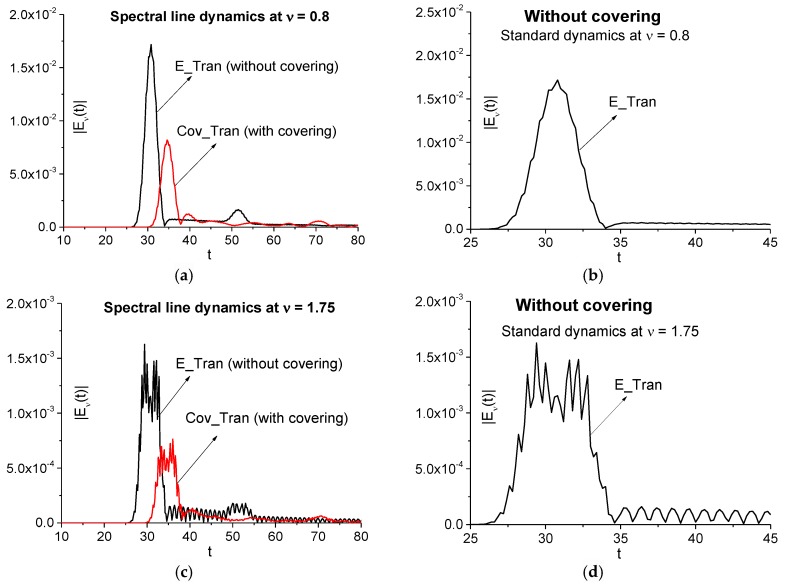
Spectral line dynamics at the frequencies *ν* = 0.8 (**a**), 1.75 (**c**) for the signals transmitted through the medium without covering (E_Tran) and with covering (Cov_Tran) in the time interval *t* = [10, 80] (**a**), (**c**); [25, 45] (**b**), (**d**).

Figure 18a,c show the evolution of spectral intensity at the frequencies *ν* = 0.8, 1.75 for the signals transmitted through the medium without covering (E_Tran) and with it (Cov_Tran) during the time interval *t* = [10, 80]. As in the case of a two energy-level medium (see Figure 8a,b), one can observe the time delay between spectral dynamics in (a), (c), which is caused by the optical density of the covering. We see also two times decreasing in maximal spectral intensities of the Cov_Tran dynamics in (a), (c). It should also be noted that the different shape of the spectral dynamics corresponding to the absorption frequency *ν* = 0.8 in (a) and the emission frequency *ν* = 1.75 in (c). Using the ICC, we have to calculate the spectral line dynamics of the signal transmitted through the non-covered medium (E_Tran) at the frequencies *ν* = 0.8, 1.75 in the time interval containing the main pulse only (b), (d).

**Figure 19:**
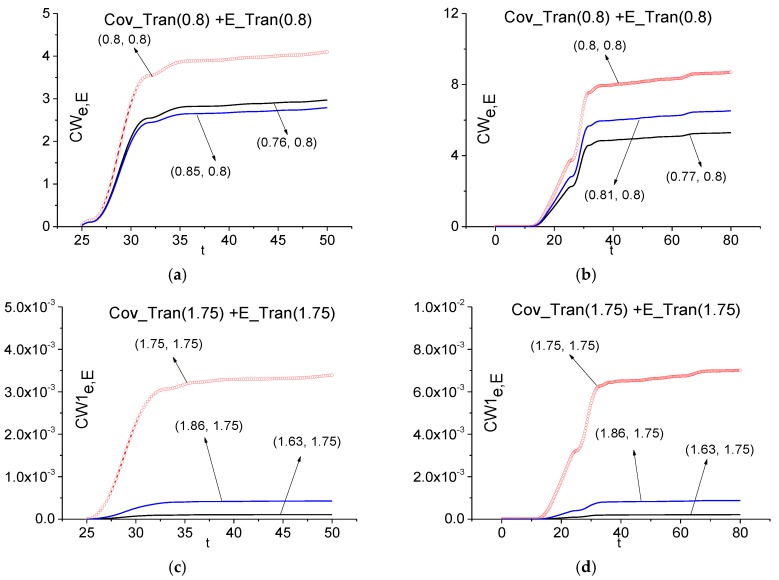
Time-dependent ICC *CW_e,E_* (**a**), (**b**), *CW1_e,E_* (**c**), (**d**) calculated at the frequencies *ν* = 0.8 (**a**), (**b**), 1.75 (**c**), (**d**) in the time intervals *t* = [25, 50] (**a**), [0, 80] (**b**) for the signal transmitted through the medium with covering (Cov_Tran) and the standard signal transmitted through the non-covered medium (E_Tran).

First, we consider the spectral features of the signal transmitted through the medium with covering (Cov_Tran) by means of the ICC *CW_e,E_* and *CW1_e,E_*. In Figure 19a,b the ICC *CW_e,E_* is calculated at the absorption frequency *ν* = 0.8 in the time interval *t* = [25, 50] (a) containing the main pulse and interval *t* = [0, 80] containing the full signal. The corresponding FDR are *ν* = [0.76, 0.85] (a) and [0.77, 0.81] (b). We see that in both cases (a), (b) the frequency *ν* = 0.8 is detected as the absorption frequency belonging to the uncovered medium. This result is important in practice, since it shows the possibility of detecting the substance absorption frequency even in a highly noisy signal, in which the main pulse amplitude is comparable with the noise amplitude.

In (c), (d) we use a medium emission frequency for using the ICC *CW1_e,E_*. One can detect the frequency *ν* = 1.75 as an emission frequency of the uncovered medium both in the time interval *t* = [25, 50] containing the main pulse and the long-time interval *t* = [0, 80], containing the full signal Cov_Tran. We see very good contrast of these lines because a generation of other used frequencies occurs with delay. Moreover, one can use for the detection very short time interval (Figure 19c,d). Of course, this time interval depends on relaxation time of the excited energy level. In contrast to the case (a), (b), the FRD for the detection of the emission frequency *ν* = 1.75 is not changed with the time interval increasing. Therefore, the high emission frequency *ν* = 1.75 using is more resistant to the change of the time interval length.

Thus, use of the criteria *CW_e,E_* and *CW1_e,E_* allows us to detect the spectral features of the non-covered three energy-level medium in the signal transmitted through the medium covered with disordered structure (Cov_Tran) in the time interval containing the main pulse *t* = [25, 50] as well as in the long-time interval *t* = [0, 80]. It should be emphasized that the detection contrast is greater at using the emission frequency *ν* = 1.75 despite its low spectral intensity.

#### 3.3.2. Reflected Signals

**Figure 20:**
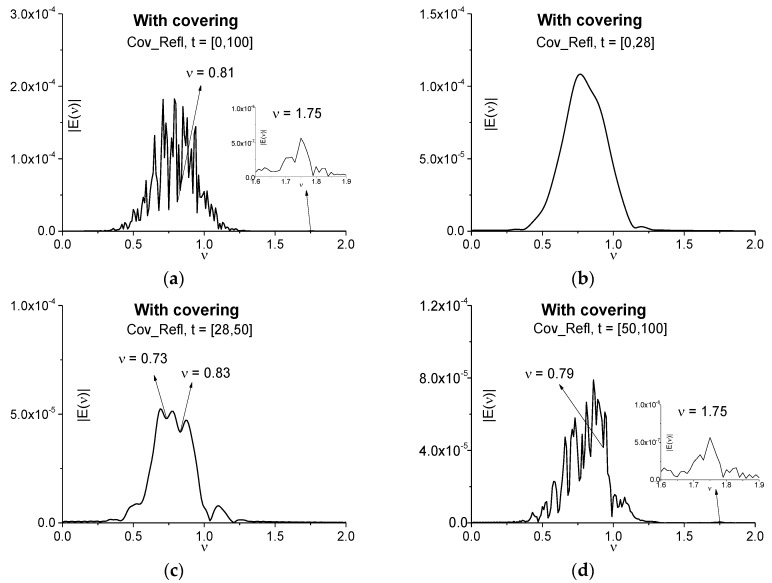
Fourier spectra of the signal reflected from the medium with covering (Cov_Refl) and calculated in the time intervals *t* = [0, 100] (**a**), [0, 28] (**b**), [28, 50] (**c**), [50, 100] (**d**); spectral resolution is Δν = 0.01.

In this section, we investigate a possibility of substance detection using the pulse reflected from the three energy-level medium with and without covering if the medium response is non-resonant. As in the Section 3.3.1, the parameter χ [37] is equal to 3. This parameter obviously refers to the Fresnel reflection from the medium boundaries and plays the definite role in the formation of reflected pulses. Figure 20 shows the Fourier spectra of the signal reflected from the medium with covering (Cov_Refl) and calculated in different time intervals: *t* = [0, 100] (a), [0, 28] (b), [28, 50] (c) and [50, 100] (d) to clarify when we obtain the highest efficiency of the detection. Spectral resolution is equal to Δν = 0.01 for all cases (a)–(d). Time interval (a) contains the full reflected signal Cov_Refl; time interval (b) contains the main pulse only; time intervals (c) and (d)—the second (c) and the last several subsequent sub-pulses (d). The spectrum of the full reflected signal Cov_Refl (a) contains the minimum at the frequency *ν* = 0.81, close to the energy-level transition frequency *ν* = 0.8 of the non-covered medium. Nevertheless, we see a strong modulation of the spectrum. As usual, there is no minima close or equal to *ν* = 0.8 in the Cov_Refl main pulse spectrum (b). One can see minima at the frequency *ν* = 0.83 in (c) and at *ν* = 0.79 in (d), which are also close to the emission frequency of the medium without covering. Apparently, the frequency shift in (a), (b), (c) may be caused by the influence of covering. However, in any cases we see the strong spectrum modulation.

Therefore, shifting of the absorption frequency and a strong modulation of the reflected signal spectrum leads to the necessity of the ICC using for the detection and identification of the substance. But it is very important that the emission intensity maximum at the frequency *ν* = 1.75 is observed in the full signal spectrum (a) as well as in the spectrum of the reflected signal in the time interval *t* = [50, 100] (d). Thus, it can be used additionally for the detection and identification of a substance by using the reflected signal spectrum.

**Figure 21:**
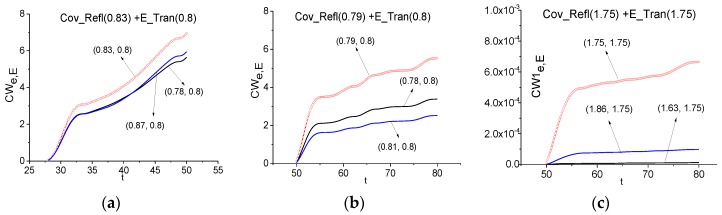
Time-dependent ICC *CW_e,E_* (**a**), (**b**) and *CW1_e,E_* (**c**) calculated for the frequencies *ν* = 0.83 (**a**), 0.79 (**b**), 1.75 (**c**) in the time intervals *t* = [28, 80] (**a**), [50, 100] (**b**), (**c**) for the signal reflected from the covered medium (Cov_Refl) and the standard signal transmitted through the non-covered medium (E_Tran).

Taking into account the reasons mentioned above, in Figure 21 the time-dependent ICC *CW_e,E_* is calculated at the frequencies *ν* = 0.83 (a), 0.79 (b) in the time intervals *t* = [28, 50] (a), [50, 100] (b), which do not contain the Cov_Refl main pulse because it does not contain information about the absorption and emission frequencies. In both cases the frequencies *ν* = 0.83 (a), 0.79 (b) are detected as the absorption frequencies of the non-covered medium in the Cov_Refl signal. It means that despite the absorption frequency shifting, a shape of the spectral line dynamics at these frequencies remains the same. That is why we can use these frequencies for the identification of a substance.

A new possibility is demonstrated in Figure 21c dealing with the time interval *t* = [50, 100] containing the remote part of the reflected signal Cov_Refl with sub-pulses. We see in (c) that using the ICC *CW1_e,E_* allows one to detect the frequency *ν* = 1.75 as the emission frequency of the uncovered medium. As in the case of the transmitted signal Cov_Tran, the detection contrast in (c) is greater for the emission frequency *ν* = 1.75 than for the frequency *ν* = 0.79 (b). Moreover, any shifting of this frequency is absent.

Therefore, in the case of the three energy-level medium the ICC *CW_e,E_* and *CW1_e,E_* allow the detection of the absorption frequency of the uncovered medium both in the spectrum of the main pulse and in spectrum of the full signal transmitted through the medium with covering (Cov_Tran). At using the reflected signal for substance detection, the spectral feature of the non-covered medium is detected at shifted frequencies contained in the time intervals not containing the main pulse. The emission frequency is not shifted by disordered structure and it is detected using the sub-pulse of the reflected signal Cov_Refl. Its detection contrast is essentially greater than for the absorption frequencies.

## 4. Conclusions

In the present paper, we demonstrate the false absorption frequencies appearing in the spectra of the pulses transmitted through or reflected from a medium covered with a disordered layered structure. As an example of such a structure in real life, we present the sample with thin paper napkins and show the inefficiency of the standard THz-TDS method for substance detection and identification under real conditions because it detects the presence of many dangerous substances (explosives and illicit drugs) in a neutral substance.

To explain the physical mechanism for the false absorption frequencies appearance in the THz signal transmitted through or reflected from the multilayered sample, we made a computer simulation using the 1D Maxwell’s equations and matrix density formalism for a medium description. In the present paper, we consider two- and three-energy layer medium at the resonant (*χ* = 0) as well as at both resonant and non-resonant (*χ* = 3) interaction of a THz pulse with this medium.

The analysis of the Fourier spectra of the averaged transmitted and reflected signals in the case of two- and three-energy layer medium shows that the false absorption frequencies do not save their positions in the dependence of number of random realizations and number of spectra minima decreases with increasing the average number. This is additional argument proving that the appearance of these frequencies in the spectra of transmitted signals is due to the disordered structure influence and it is possible to use this averaging for the false absorption frequencies removal from an analysis of the spectral properties of the substance.

In the case of three-energy layer medium (*χ* = 3) the high emission frequencies appearing due to the cascade mechanism of high energy level excitation for both transmitted and reflected signals is demonstrated. The disordered cover does not distort these frequencies.

The spectral characteristics of the transmitted and reflected signals in both cases are analyzed by means of the SDA-method and several ICC’s with the aim of substance detection and identification.

In the transmitted signal the ICC’s detect the absorption and emission frequencies of the non-covered medium both in the main pulse and in the long time interval, containing the full transmitted signal. In the reflected signal the absorption and emission frequencies of the non-covered medium are detected (with slight shift) in the time interval containing the first sub-pulse and in the remote part of the signal. We do not use for substance detection and identification the main pulse of the reflected signal because it does not contain information about the absorption and emission frequencies.

The disordered structure distorts the high emission frequency less in comparison with a lower absorption frequency. Thus, the cascade mechanism may provide possibilities for the detection of some dangerous substances (HMX, drugs MA, MDA), which have no pronounced absorption frequencies in the spectra, for example. The emission frequency using may be effective tool for the detection and identification of substances based on integral correlation and likeness criteria.

The absence of dangerous substance (for example, RDX) in the medium covered by disordered structure, were demonstrated also by means of ICC.

It should be stressed that integral correlation and likeness criteria allow us to detect the spectral feature of the standard medium in the remote part of the reflected and averaged signals, which does not contain the main pulse. This result is important for practice, since it shows the possibility of detecting the substance absorption or emission frequency even in a highly noisy signal, in which the main pulse amplitude is comparable with the noise amplitude.

In order to enhance the detection reliability we propose to use several integral criteria simultaneously. The method discussed is a promising and competitive tool for the reliable detection and identification of various substances both in real and laboratory environments in comparison with the THz TDS method, based on the comparison of substance spectra. The method can be used for security screening applications, for non-destructive testing, as well as for quality control in the pharmaceutical and food industry.

## Abbreviations

The following abbreviations are used in this manuscript:

THz TDS	terahertz time-domain spectroscopy
SDA-method	Spectral Dynamics Analysis method
ICC	integral correlation criteria
FDR	frequency detection range
